# The Classification of the Insane

**Published:** 1899-03-18

**Authors:** 


### THE CLASSIFICATION OF THE INSANE.

What's in a name? If the opinion of many of the inhabitants of our public asylums and their relatives was asked, it would be found that they have, and with reason, great objections to be classed as paupers. It is unfair and impolitic that they should be so styled. During recent years the nursing of the insane has been much improved, and in most institutions the staff now undergo a course of training in their duties. Magnificent buildings are provided to act as hospitals for the acutely insane, and as repositories where human wreckage is cared for. In so lavish a way are some of these institutions furnished that the cynical have described them as palaces for paupers. No member of society is, comparatively speaking, so carefully watched over as the epileptic in one of our public asylums. He is seen three times a day by a medical man. Every mark or slight injury he may receive in a fit is noted and inquired into. He is constantly under the supervision of skilled attendants, and even in his slumbers is carefully tended.

Though the friends of the insane poor begin to recognise the solid benefits their relatives receive in our public asylums, they resent their being classed as paupers. Is there any necessity to stigmatise them thus? It is true that the charge is paid for through the guardians, but in many cases the friends repay to them the full amount expended from the poor rate. It has been decided of late in the case of some pensioners that when the pension covers the amount charged for care and maintenance the pensioner is to be looked upon as technically a private patient. The amount thus received only covers the weekly charge for maintenance, and no contribution is received from them towards the cost of keeping up the fabric of the asylum. Such cases are, therefore, not on the same level as an ordinary private patient, but still it is hardly just to class them as paupers. If then, a distinction is to be made in the case of these pensioners, why not also in the case of the respectable poor whose friends repay all that is expended by the guardians?

Every obstacle, sentimental or otherwise, which hinders a lunatic being placed under treatment at the earliest possible stage of his illness, and which causes unnecessary irritation when under treatment should, as far as possible, be minimised. With many the idea that their relatives would be classed as paupers if sent to an asylum does deter them from sending the patient until compelled to do so, while many of the patients themselves recognise their position and resent it. It is a pity that some means have not been devised to remove this grievance, which, though but a sentimental one, has an appreciable influence.

The next point is one which is already occupying some attention. Why should the respectable insane be compelled to associate with the criminal lunatic? It is bad enough that they are classed as paupers, though this may not carry with it any practical hardship. The enforced association with individuals whose proper place is in the wards of a criminal lunatic asylum is, however, anything but a fanciful grievance. Some further provision ought to be made for the reception of criminal lunatics. It is not right that they should be sent into the wards of a county or borough asylum.

